# Phosphatidylserine Exposed Lipid Bilayer Models for Understanding Cancer Cell Selectivity of Natural Compounds: A Molecular Dynamics Simulation Study

**DOI:** 10.3390/membranes12010064

**Published:** 2022-01-01

**Authors:** Navaneethan Radhakrishnan, Sunil C. Kaul, Renu Wadhwa, Durai Sundar

**Affiliations:** 1 DAILAB, Department of Biochemical Engineering and Biotechnology, Indian Institute of Technology (IIT) Delhi, New Delhi 110016, India; navaneethan@dbeb.iitd.ac.in; 2 AIST-INDIA DAILAB, DBT-AIST International Center for Translational and Environmental Research (DAICENTER), National Institute of Advanced Industrial Science and Technology (AIST), Tsukuba 305-8565, Japan; s-kaul@aist.go.jp; 3 School of Artificial Intelligence, Indian Institute of Technology (IIT) Delhi, New Delhi 110016, India

**Keywords:** phosphatidylserine, cancer cells, MD simulation, membrane permeability, withaferin A, withanone, CAPE, artepillin C

## Abstract

Development of drugs that are selectively toxic to cancer cells and safe to normal cells is crucial in cancer treatment. Evaluation of membrane permeability is a key metric for successful drug development. In this study, we have used in silico molecular models of lipid bilayers to explore the effect of phosphatidylserine (PS) exposure in cancer cells on membrane permeation of natural compounds Withaferin A (Wi-A), Withanone (Wi-N), Caffeic Acid Phenethyl Ester (CAPE) and Artepillin C (ARC). Molecular dynamics simulations were performed to compute permeability coefficients. The results indicated that the exposure of PS in cancer cell membranes facilitated the permeation of Wi-A, Wi-N and CAPE through a cancer cell membrane when compared to a normal cell membrane. In the case of ARC, PS exposure did not have a notable influence on its permeability coefficient. The presented data demonstrated the potential of PS exposure-based models for studying cancer cell selectivity of drugs.

## 1. Introduction

Cancer cells are highly complex and extremely difficult to treat due to the involvement of multifactorial signaling pathways involved in the process of carcinogenesis. These involve genetic and somatic aberrations that are highly heterogeneous, often leading to intra-tumor heterogeneity. Molecular targeted therapies use small molecules to target one or more proteins involved in the regulation of cell cycle progression, cancer signaling pathways, angiogenesis, growth arrest and/or apoptosis in cancer cells. By activating or inhibiting the target proteins, such small molecules/drugs block cancer cell proliferation and tumor growth. Many of these small molecules can also affect cell migration and invasion capability, and therefore are used for blocking cancer metastasis. However, most cancer drugs have very low therapeutic indices and are used near their maximum-tolerated doses to attain clinically meaningful results [1]. Adverse side effects of chemotherapeutic drugs pose a major concern in cancer chemotherapy [2]. These can be attributed to their effect on normal cells, due to low or lack of selectivity to cancer cells [1]. Hence, there is a need to develop innovative strategies to predict and measure the cancer cell selective effects of chemotherapeutic drugs. 

Interactions of drugs with the cell membrane are critical, as the drugs must cross the lipid bilayer of the cells to reach their intra-cellular targets. Malignant transformation has been shown to involve alterations in the lipid profile of cell membranes [3,4]. Changes in the levels of different types of lipid molecules in cell membranes have been reported in various types of cancers [5,6,7,8,9,10]. A common hallmark observed across several types of cancers appears to be the loss of asymmetry in the distribution of different types of lipid molecules between the two leaflets of the cell membrane [11,12,13]. The basic structure of the cell membrane consists of a lipid bilayer that is mainly composed of phospholipids. Phosphatidylcholines (PC), phosphatidylethanolamines (PE), phosphatidylserines (PS), phosphatidylinositols (PI) and sphingomyelins (SM) form the majority of the phospholipids in the cell membrane [14]. A non-uniform distribution of these lipids across the two leaflets of the bilayer is a characteristic feature of a normal eukaryotic cell membrane [14,15]. PS and PE are usually present in the inner leaflet, while the outer leaflet is mostly composed of PC and SM [14,15]. This asymmetric distribution is actively maintained by the ATP-dependent enzymes, flippases and floppases [16]. PS and PE are transported from outer leaflet to inner leaflet by flippases, while PC and SM are transported in the opposite direction by floppases [16]. An absence of such asymmetric distribution of phospholipids has been reported in cancers. Exposure of phosphatidylserine and phosphatidylethanolamine molecules on the outer leaflet has been reported in cancer cells [11,12,13]. The altered distribution of lipids in cancer cell membranes makes their structural and biophysical properties different to that of normal cells. Such changes could modulate drug penetration, thereby influencing drug activity [4].

Molecular dynamics simulations using atomistic models of lipid bilayers made up of PC, SM, PS, PE and/or cholesterol have been used by different studies to investigate the effects of asymmetric lipid distribution on membrane properties [17,18,19,20]. Studies on the permeability of lipid bilayers to small molecules using molecular dynamics simulations have demonstrated the potential of in silico membrane models in analyzing the membrane permeation of drugs [21,22,23,24]. 

In the present study, molecular dynamics simulations involving in silico atomistic models of lipid bilayers have been used to explicitly explore the effect of PS exposure in cancer cells on membrane permeation of natural compounds reported to have anti-cancer properties. Atomistic lipid bilayer models of cancer and normal cell membranes used in this study are based on the relative distribution of two kinds of phospholipids: the most common phospholipid, phosphatidylcholine (PC), and the anionic phospholipid which is exposed exclusively in cancer cells, phosphatidylserine (PS). Both the bilayer models were built using PC and PS in the ratio 2:1 that roughly equates to their reported proportion [14]. The normal cell membrane model was built to have all the 1-palmitoyl-2-oleoyl-sn-glycero-3-phosphoserine (POPS) molecules in the inner leaflet, whereas the cancer cell membrane model was built with POPS molecules in both leaflets of the membrane. For simplicity, other types of phospholipids and sterols were not included in the model. 

**Figure 1:**
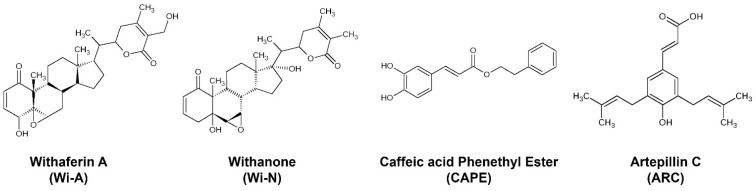
The 2D pictorial representation of natural compounds: Withaferin A (Wi-A), Withanone (Wi-N), Caffeic Acid Phenethyl Ester (CAPE) and Artepillin C (ARC).

The natural anti-cancer compounds chosen for this study included Withaferin A (Wi-A), Withanone (Wi-N), Caffeic Acid Phenethyl Ester (CAPE) and Artepillin C (ARC) (Figure 1). These are bioactive molecules from natural sources known for their therapeutic potential with lesser side effects compared to synthetic drugs. Wi-A and Wi-N are secondary metabolites from Ashwagandha that have been extensively studied for their anti-cancer activities [25,26,27,28]. Molecular modeling approaches accompanied with in vitro assays have revealed the multi-modal anti-cancer activities of Wi-A and Wi-N [25,26,27,29]. Although Wi-A and Wi-N are closely related structural analogs, several previous studies have reported their different levels of cytotoxicity in cancer and normal cells [30,31]. Whereas Wi-A showed stronger cytotoxicity to both cancer and normal cells, Wi-N exhibited milder toxicity to cancer cells and was safer for normal cells [31]. CAPE, a bioactive compound isolated from New Zealand honeybee propolis, was earlier studied for its anti-cancer activities and reported to cause the death of cancer cells selectively [26]. Another propolis-derived bioactive compound, ARC—particularly enriched in Brazilian honeybee propolis—has also been reported for its anti-cancer activity [32,33,34]. 

In this study, we examined the permeation of Wi-A, Wi-N, CAPE and ARC using the models of cancer and normal cell membranes, with particular reference to the effect of PS exposure in cancer cells. Computational free energy profiles of drugs across the membrane provide a good understanding of their permeation mechanisms [35,36]. Potential of mean force (PMF) values derived through molecular dynamics simulations demonstrate the free energy landscape of permeation of drugs through the membrane. Classical molecular dynamics simulations are not suitable for efficiently sampling the configuration space for PMF calculations, as the systems might remain trapped in local free energy minima leaving out the events involving large energy barriers. Umbrella sampling methods coupled with molecular dynamics simulations have been proven to be well suited for generating PMF profiles [37]. Steered molecular dynamics (SMD) simulations, in which an external force is applied to an atom of the drug molecule to pull the molecule through the membrane, can be used for generating initial configurations for umbrella sampling. SMD simulations and umbrella sampling methods have been successfully used by different studies for deriving the PMF profiles of lipid membrane traversal of the small molecules [22,23,38,39,40]. Here, SMD and umbrella sampling simulations were employed to derive the effect of PS exposure in the cancer membrane on the permeation of chosen molecules.

## 2. Materials and Methods

### 2.1. Generation of Lipid Bilayer Systems

Two atomistic lipid bilayer systems were generated using CHARMMGUI [41]. Each of the generated systems contained 48 molecules of POPC (1-palmitoyl-2-oleoyl-sn-glycero-3-phosphocholine), 24 molecules of POPS (1-palmitoyl-2-oleoyl-sn-glycero-3-phosphoserine) and ~5000 water molecules. Table 1 shows the distribution of lipids in the leaflets of the membrane. NaCl was used to neutralize the system and an additional 0.15 M NaCl was added to maintain the salt concentration.

### 2.2. Equilibration of Lipid Bilayer Systems

Classical molecular dynamics simulations were used for equilibrating the generated cancer and normal membrane systems. CHARMM36 force field parameters were used for all molecules in the systems [42]. TIP3P water model was used. All molecular dynamics simulations were performed in GROMACS 2020 using leap-frog integrator and Verlet cutoff scheme [43]. PME method with a cut-off distance of 1.2 nm was used for calculating coulomb interactions [44]. The systems were periodic in all directions.

Energy minimization was performed for 5000 steps using steepest descent algorithm. During energy minimization, position restraints were applied to phosphate atoms with a force constant of 1000 kJ/mol/nm^2^. After energy minimization, simulation was conducted in canonical (NVT) ensemble for 100 ps with a timestep of 1 fs, during which a temperature of 310 K was reached using a velocity rescale thermostat with a time constant of 1 ps. During NVT equilibration, position restraints were applied to phosphate atoms with a force constant of 1000 kJ/mol/nm^2^.

After NVT equilibration, the systems were equilibrated in isothermal–isobaric (NPT) ensemble in six stages: from NPT-1 to NPT-6 for 502.5 ns. Position restraints applied to the phosphate atoms were gradually relieved during NPT-1 to NPT-4, with no restraints applied in NPT-5 and NPT-6. The force constants used for position restraints on phosphate atoms were 600 kJ/mol/nm^2^, 400 kJ/mol/nm^2^, 200 kJ/mol/nm^2^ and 50 kJ/mol/nm^2^ from NPT-1 to NPT-4, respectively. Timestep was set to 1 fs in NPT-1 and NPT-2, while from NPT-3 onwards it was set to 2 fs. From NPT-1 to NPT-5, temperature was maintained at 310 K using a velocity rescale thermostat with a time constant of 1 ps, while pressure was maintained at 1 atm by semi-isotropic coupling using Berendsen barostat with a time constant of 5 ps. During NPT-6, Nose–Hoover thermostat with a time constant of 1 ps and Parrinello–Rahman barostat with a time constant of 5 ps were used to maintain the temperature and the pressure at 310 K and 1 atm, respectively. NPT-1 to NPT-6 were run for a duration of 250 ps, 250 ps, 1 ns, 1 ns, 300 ns and 200 ns, respectively. The production simulation during which membrane properties were assessed was run in NPT ensemble for 200 ns. No position restraints were used during production and the time step was 2 fs. Thermostat and barostat used were the same as for the NPT-6 equilibration. Snapshots were saved every 10 ps for analysis.

### 2.3. Calculation of Membrane Properties

Area per lipid and order parameters of lipid tails were calculated using MEMBPLUGIN through VMD [45,46]. Area per lipid was computed by selecting a triad of atoms for phospholipids, projecting their x and y coordinates into a plane, dividing them into polygons using a Voronoi diagram [47] and then calculating the area of the polygons. The formula used for order parameter (*S_CD_*) calculation is as follows [48]:

(1) $$S_{\mathrm{CD}}=-\frac{1}{2}{\;3\;\cos}^{2}\theta \;-\;1,\;$$

where *θ* is the instantaneous angle between the C–H bond and the bilayer normal.

### 2.4. Steered Molecular Dynamics Simulations and Umbrella Sampling

To compute PMF and diffusivities of the molecules, configurations and energies of drug molecules had to be sampled along the bilayer normal. Steered molecular dynamics simulations were performed to generate the initial configurations for sampling. The force field parameters of small molecules Wi-A, Wi-N, CAPE and ARC were generated using CGENFF server [42].

The small molecule was inserted into the water at around 2 nm from the lipid head groups. After insertion of the small molecule into the system, energy minimization was performed using steepest descent algorithm for 5000 steps, followed by NVT and NPT equilibration for 50 ps and 100 ps, respectively. Position restraints were applied to the small molecule in Z-direction during NVT and NPT equilibration. Steered MD simulations were performed in NVT ensemble using the pull-code of GROMACS. Nose–Hoover thermostat with a time constant of 1 picosecond and Parrinello–Rahman barostat with a time constant of 5 ps were used to maintain the temperature and pressure at 310 K and 1 atm, respectively, during the pulling simulation. The small molecule was pulled along Z-direction from water, towards the hydrophobic core of the membrane, using an umbrella potential with a force constant of 100 kJ/mol/nm^2^ and a pulling rate of 0.00025 nm/ps. After pulling, configurations were sampled every 0.2 nm along the reaction coordinate: the Z-component of distance (*z*). A total of 22 windows spaced 0.2 nm apart were taken for umbrella sampling (US). 

For each window, US simulations were performed in NPT ensemble, with a harmonic force constant of 100 kJ/mol/nm^2^. Nosé–Hoover thermostat was used for maintaining temperature at 310 K, with a semi-isotropic Parrinello–Rahman barostat used to maintain pressure at 1 atm. Each window was run for 40 ns, and data from the last 25 ns were used for analysis. Positions and forces along the reaction coordinate were saved every 2 fs. For the cancer cell membrane, US simulations were performed along only one leaflet, as the membrane has a symmetric distribution of lipids between the two leaflets. For the normal cell membrane, US sampling simulations were performed along both leaflets independently.

### 2.5. Calculation of PMF and Permeability Coefficients

PMF profiles were estimated using Weighted Histogram Analysis Method (WHAM) implementation in GROMACS [27]. For the normal membrane model, PMF values were calculated for both leaflets separately, starting from bulk water to membrane core, and the PMF curves were smoothened by using moving average spanning 0.5 Å. In the case of the cancer membrane, PMF values of one leaflet were duplicated for the other, with moving average smoothing conducted the same way as for the normal membrane. The convergence of PMF profiles was confirmed by checking PMF values at different intervals of umbrella sampling simulations (Supplementary Materials: Figure S1). The permeability coefficient (*P*) was calculated using the Inhomogeneous Solubility Diffusion model [35,36]. Accordingly, *P* was derived from effective resistivity (*R_eff_*) using the relation,

(2) $$P=\frac{1}{R_{\mathrm{eff}}},\;$$

and *R_eff_* was calculated using,

(3) $$R_{\mathrm{eff}}=\int_{z_{1}}^{z_{2}}R\left(z\right)\;\mathrm{dz},$$

where *z* is a collective variable describing the relative position of the solute along the reaction coordinate and *R*(*z*) is the resistivity at *z*. *R*(*z*) was calculated from free energies (Δ*G*(*z*)) and diffusion coefficients (*D*(*z*)) along *z* using the equation,

(4) $$R\left(z\right)=\;\frac{{e\;}^{\beta \Delta G(z)}}{D(z)}\;$$

where *β* is the inverse of the Boltzmann constant times the temperature. 

Diffusion coefficients were calculated using the method proposed in 2005 by Hummer [49], from the autocorrelation function (*C_zz_*(*t*)) of *z* and variance (*var*(*z*)) of *z* as follows.

(5) $$D\left(z\right)=\frac{{\mathrm{var}(z)}^{2}}{\int_{\;0}^{\;\infty }C_{\mathrm{zz}}\left(t\right)\;\mathrm{dt}}\;$$

*C_zz_*(*t*) was calculated using the ”analyze” module of GROMACS.

## 3. Results and Discussion

**Figure 2:**
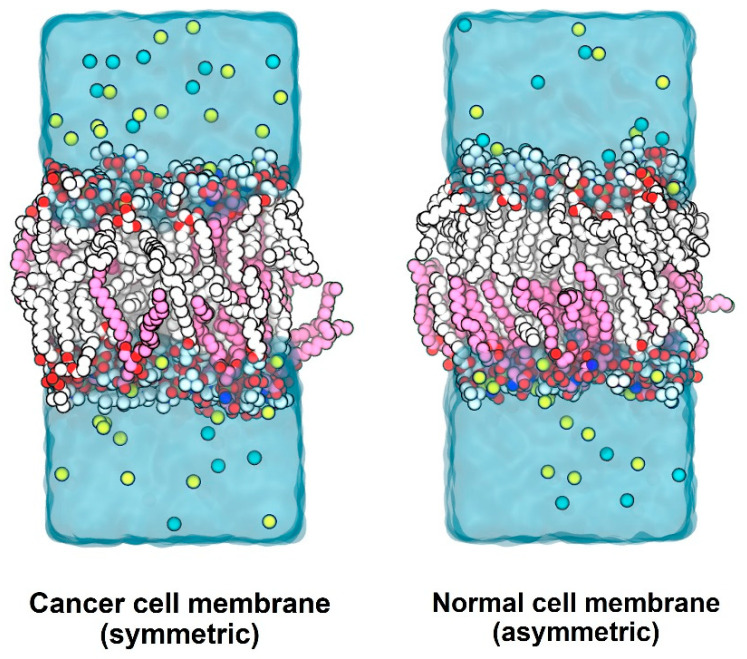
Equilibrated bilayer systems: Carbon atoms of POPC are shown in white, carbon atoms of POPS in magenta, sodium atoms in yellow and chloride atoms in cyan, with hydrogen atoms not shown. The outer (extracellular) leaflet is at the top and the inner (cytoplasmic) leaflet at the bottom. The normal cell membrane model contains POPS only in the inner leaflet, whereas the cancer cell membrane model contains POPS in both leaflets.

The phospholipid PS is usually present in the inner leaflet of the cell membranes of normal cells [14,15]. Exposure of PS on the cell membrane of cancer cells has been reported in a wide range of cancers [11,12,50,51]. Here, atomistic models of cancer and normal membranes based on a relative PS distribution between the outer and the inner leaflets of the membrane were built to study the permeation of some natural compounds (Wi-A, Wi-N, CAPE and ARC) shown to possess anti-cancer activity. The built membrane models consisted of only two types of lipid molecules, POPC and POPS, in contrast to the diversity of lipid molecules in the cell membrane. Usage of such simplistic models reduced the simulation time needed for proper equilibration and production, compared to complex models. The non-inclusion of other types of lipid molecules of the biological cell membrane in our models may have some limitations. However, the models fitted the purpose of simulation, which was to study the influence of PS exposure on the membrane outer surface on drug permeation. Simplistic models composed of one/two kinds of lipid molecules have been conservatively used in the past for drug–membrane interaction studies and have been shown to produce results comparable with experimental studies [52]. The lipid bilayer systems were equilibrated for roughly 500 ns and production simulations were run for 200 ns prior to introducing drug molecules into the systems. Figure 2 shows the cancer and normal lipid bilayer systems after equilibration. Different properties of the lipid membranes were computed during the production runs, to check if the membranes were adequately equilibrated.

### 3.1. Structural Properties of Lipid Bilayer Systems

**Figure 3:**
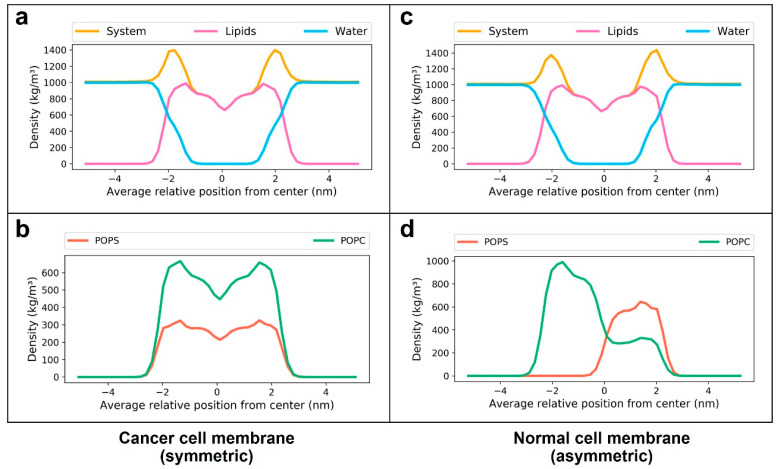
Densities of components of the equilibrated membrane systems: (**a**,**b**) cancer cell membrane and (**c**,**d**) normal cell membrane. The densities of the whole system (yellow), lipids (magenta) water (cyan), POPC (green) and POPS (orange) along the reaction coordinate “*z*” are shown. On the horizontal axis, the value 0 indicates the center hydrophobic core of the membrane, negative values indicate the outer (extracellular) leaflet and positive values indicate the inner (cytoplasmic) leaflet.

**Figure 4:**
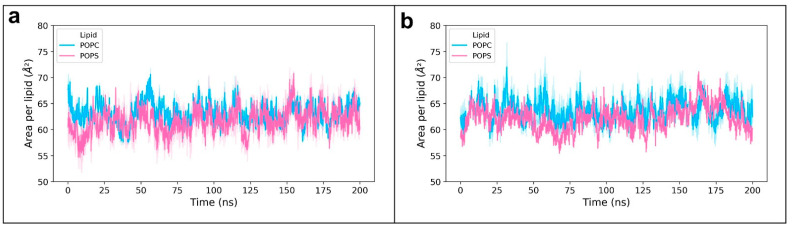
Time-dependent area per lipid values of the membrane models: (**a**) normal membrane and (**b**) cancer membrane. POPC (cyan) and POPS (magenta).

**Figure 5:**
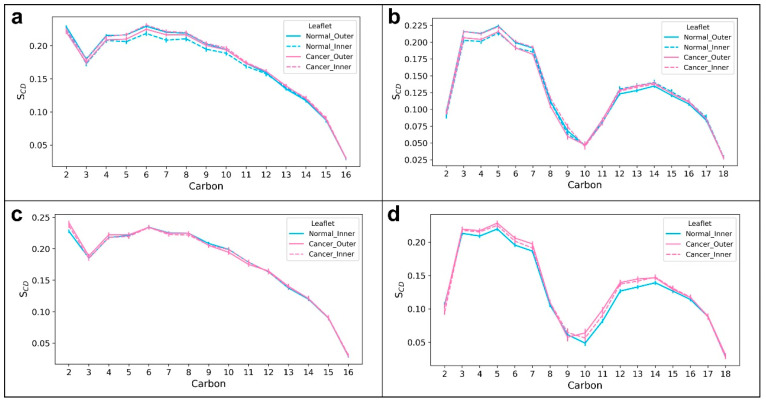
Order parameters of hydrophobic tails of lipid molecules in equilibrated systems: (**a**) POPC sn1, (**b**) POPC sn2, (**c**) POPS sn1 and (**d**) POPS sn2. The outer and inner leaflets of a normal membrane are shown in solid cyan and dashed cyan lines, respectively. The outer and inner leaflets of a cancer membrane are shown in solid magenta and dashed magenta lines, respectively.

Densities of different system components across the bilayer normalwere measured to verify the localization of different molecules in the systems. The densities of different components of the systems along the direction of normal to the membranes (Z-direction) are shown in Figure 3. On the horizontal axis, “0” indicates the center of the hydrophobic core of the membrane, while negative values point towards the outer leaflet and positive values point towards the inner leaflet. The peaks at “−2” and “+2” indicate the polar heads of lipid molecules. Figure 3d shows the asymmetric distribution of POPC and POPS molecules in the normal membrane in contrast to the symmetric distribution in the cancer membrane (Figure 3b). The area per lipid denotes the average cross-sectional area of lipid molecules in the membrane leaflets. Time evolution of the average area per lipid is a good criterion to check if the system has reached a steady state [53]. The time-dependent area per lipid of the leaflets of cancer and normal membranes is shown in Figure 4. As there are no noticeable drifts in area per lipid over the course of simulation, it was assumed that the bilayers were stable. Lipid-tail-order parameters are a measure of alignment of lipid tails with respect to the bilayer normal. The use of order parameter profiles is a widely used method for characterizing the structure of hydrocarbon region in lipid bilayers. Order parameters were calculated for sn1 and sn2 tails of POPC and POPS molecules in the membrane using Equation (1) described in the Section 2. Figure 5 shows the order parameters of lipid molecules in cancer and normal membranes. Order parameter plots of sn2 tails are characteristically different from that of sn1 tails because of the low order parameters around carbons 9 and 10 of sn2 tails, attributed to the presence of a double bond in the oleoyl group. The values were close to the previously reported values in the literature [20,54]. In view of these data, the membrane models were considered equilibrated enough and used for drug permeation studies.

### 3.2. PMF Profiles of the Natural Compounds

**Figure 6:**
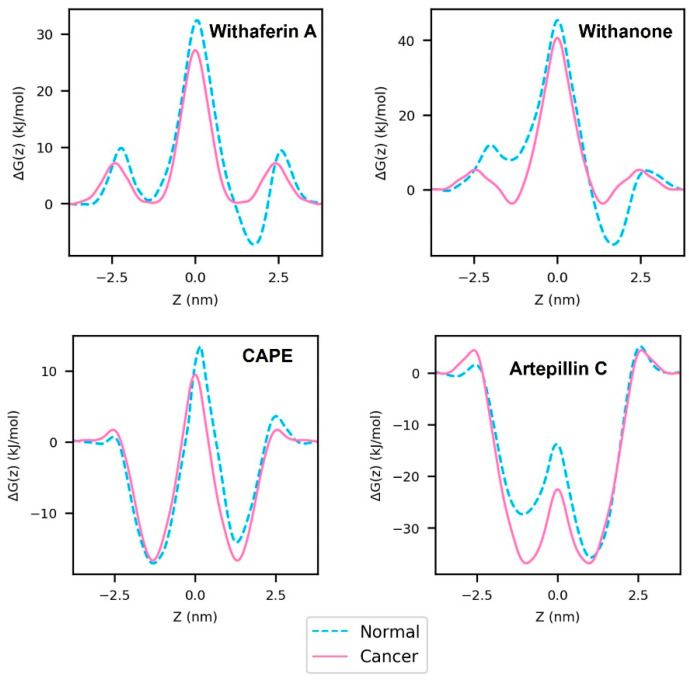
PMF profiles of Withaferin A (Wi-A), Withanone (Wi-N), Caffeic Acid Phenethyl Ester (CAPE) and Artepillin C (ARC). The solid magenta line indicates the PMF profile in the cancer cell membrane model and the dashed cyan line indicates the PMF profile in the normal cell membrane model. On the horizontal axis, the value 0 indicates the center of the hydrophobic core of the membrane, negative values indicate the outer (extracellular) leaflet and positive values indicate the inner (cytoplasmic) leaflet.

SMD and umbrella sampling simulations were used to generate the PMF profiles of the selected small molecules Wi-A, Wi-N, CAPE and ARC. The PMF profiles show how the free energy changes as a function of position of the molecule through the membrane along the direction of the bilayer normal. Here, the Z-axis is the reaction coordinate along which PMF is defined. Figure 6 shows the computed PMF curves of the small molecules. The raw PMF values, with their standard deviation before moving average smoothing, are shown in Supplementary Materials: Figure S2. The magenta solid curves in Figure 6 indicate PMF in the cancer membrane and the cyan-dashed curves indicate PMF in the normal membrane. The highest point in PMF (Δ*G_max_*) indicates the energy barrier that has to be overcome for the passage of small molecules through membranes. Wi-A, Wi-N and CAPE had Δ*G_max_* in the center of the hydrophobic core of the membranes (Figure 6). Hence, the hydrophobic core of the membranes formed the principal hindrance for the passage of these three molecules. The Δ*G_max_* values of Wi-A, Wi-N and CAPE were in the order CAPE < Wi-A < Wi-N (Figure 6), predicting that CAPE is able to traverse through the membrane easily compared to Wi-A and Wi-N. Furthermore, Wi-A showed easier traversal compared to Wi-N. Previous studies that have used atomistic models of “POPC + cholesterol” bilayers to study the permeability of Wi-A and Wi-N have reported easier permeation of Wi-A through the cell membrane compared to Wi-N [23]. Our results based on POPC + POPS models were in line with the earlier report [23]. The Δ*G_max_* of Wi-A, Wi-N and CAPE was higher in the normal membrane compared to that in the cancer membrane (Figure 6). Hence, PS exposure in the outer leaflet may have facilitated easier permeation of Wi-A, Wi-N and CAPE through the membrane by reducing the energy barrier in the hydrophobic core. In the case of ARC, Δ*G_max_* occurred at the polar head group regions of lipid molecules (Figure 6). Unlike Wi-A, Wi-N and CAPE, the lipid head groups caused the principal hindrance for ARC permeation. The magnitude of Δ*G_max_* of ARC was much lower than that of the other three drug molecules in comparison (Figure 6), predicting that ARC may traverse through the membrane easier than Wi-A, Wi-N and CAPE.

As Wi-A and Wi-N are structural analogs, they have comparable PMF landscapes in cancer and normal membrane models. They show interesting differences in PMF between *z* ≈ −2.5 and *z* ≈ −1.5 (Figure 6). It is the region along the reaction coordinate where the centers of masses of drug molecules pass from bulk water to membrane. At this cell–water interface, the difference between PMF values of Wi-N in cancer and normal membranes was prominent, while Wi-A had proximate values in cancer and normal membranes (Figure 6). Differences in PMF values in cancer and normal membranes for Wi-N indicated differential binding of Wi-N to the outer leaflet of cancer and normal membranes. Lower values of PMF in the cancer cell membrane implied that more Wi-N molecules could bind to the cancer membrane compared to the normal cell membrane. Hence, it can be inferred that the exposure of PS could potentially facilitate selective binding of Wi-N to cancer cells. The propolis compounds CAPE and ARC had dissimilar structures, as did their PMF landscapes. The hydrophobic cores of both cancer and normal membranes had a higher affinity for ARC compared to CAPE, indicated by the lowest values in its PMF profile (Figure 6). This is due to the presence of the hydrophobic diprenyl groups in its structure.

The simulation trajectories were visualized, with reference to the PMF profiles, to gain insights into the structural aspects of permeation. Snapshots of the last frame from different umbrella sampling windows shown in Figures S3–S6 in Supplementary Materials indicate the converged orientations of Wi-A, Wi-N, CAPE and ARC, respectively, along the reaction coordinate at different regions of the membrane during permeation. Figures S3–S6: subfigures B and E in Supplementary Materials show the preferred orientation of molecules inside the outer and inner leaflets of the normal membrane, respectively, associated with the troughs in the PMF curves. Figures S3–S6: subfigure H in Supplementary Materials shows the preferred orientation of molecules inside the cancer membrane. In the preferred orientation, the polar groups of the natural compounds are oriented towards the head groups of lipids, with hydrophobic groups of natural compounds oriented towards the lipid hydrocarbon region. The passage of natural compounds through the membrane core involves a flip in the orientation of natural compounds from their polar groups oriented towards the head groups of outer leaflet lipids to that of lower leaflet lipids. In the case of Wi-A, Wi-N and CAPE, the primary barrier to permeation—related to Δ*G_max_*—is associated with the traversal of their polar groups through the hydrophobic core of the membrane. In light of this fact, the hydrophobic cores of cancer and normal membranes were inspected. It was found that the lipid head polar groups of the two leaflets of cancer membrane were slightly closer to the hydrophobic core in the cancer membrane compared to that in the normal membrane (Supplementary Materials: Figure S7). This could be the basis for the lower Δ*G_max_* of Wi-A, Wi-N and CAPE in the cancer membrane. In the case of ARC, the primary barrier to permeation is associated with the passage of its hydrophobic diprenyl groups through the hydrophilic head region of lipids.

### 3.3. Resistivity Profiles of the Natural Compounds

**Figure 7:**
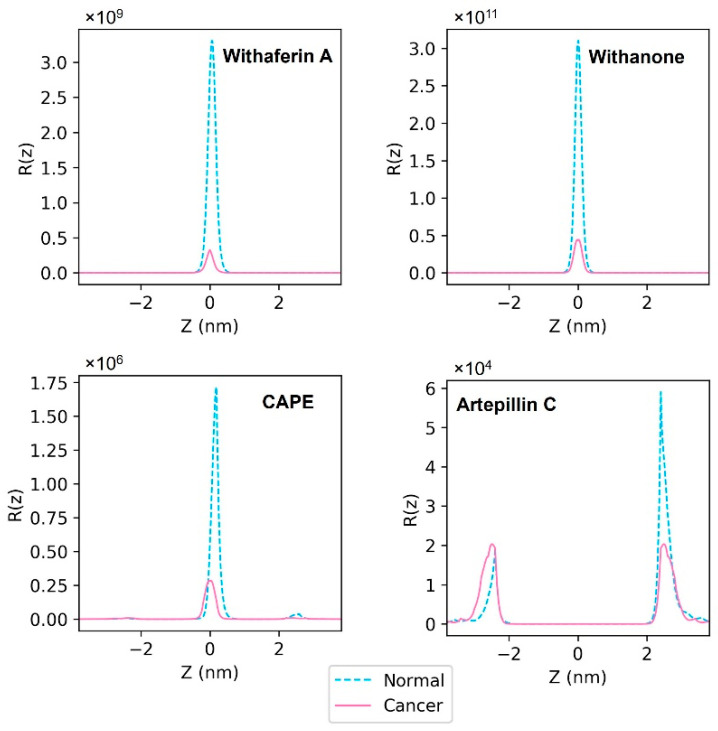
Resistivity profiles of Withaferin A (Wi-A), Withanone (Wi-N), Caffeic Acid Phenethyl Ester (CAPE) and Artepillin C (ARC). The solid magenta line indicates the resistivity profile in the cancer cell membrane model and the dashed cyan line indicates the resistivity profile in the normal cell membrane model. On the horizontal axis, the value 0 indicates the center of the hydrophobic core of the membrane, negative values indicate the outer (extracellular) leaflet and positive values indicate the inner (cytoplasmic) leaflet.

Resistivity profiles of the drug molecules were calculated from PMF (Figure 6) and diffusivity profiles (Supplementary Materials: Figure S8) using Equation 4. The calculated resistivity profiles are shown in Figure 7. The curves of Wi-A, Wi-N and CAPE were similar in shape, showing the highest resistance for permeation in the hydrophobic core of the membrane (Figure 7). The resistance offered by the hydrophobic core of the cancer membrane was less compared to that of the normal membrane for Wi-A, Wi-N and CAPE, indicating higher permeation rates in the cancer membrane. ARC had resistivity peaks at polar regions of the membrane (Figure 7). ARC had higher resistance in the inner leaflet of the normal membrane, where POPS molecules were high in number compared to that in the cancer membrane. It implied that the presence of POPS head groups increased the resistance for the passage of ARC through polar regions of the membrane.

### 3.4. Permeability Coefficients of the Natural Compounds

Permeability coefficients (P) of the drug molecules were calculated using the Inhomogeneous Solubility Diffusion model (ISD) [35,36]. Table 2 shows the permeability coefficients of Wi-A, Wi-N, CAPE and ARC in cancer and normal membranes. The permeability coefficients were compared with the computed octanol–water partition coefficients (XLogP3-AA) of the drug molecules obtained from the Pubchem database [55,56]. The octanol–water partition coefficient is a measure of lipophilicity of the molecule. The calculated permeability coefficients were directly correlated with the octanol–water partition coefficients obeying Meyer–Overton’s rule [57], confirming the validity of the methods used in our study (Table 2). The calculated permeability coefficients were in the order Wi-N < Wi-A < CAPE < ARC (Table 2). The lower permeability coefficients of Wi-A and Wi-N were anticipated due to their bigger size and the presence of polar groups all over their structures. The lower permeability of Wi-N compared to that of its analog Wi-A is in line with the higher IC50 values of Wi-N compared to that of Wi-A, as reported in previous studies (IC50 for Wi-A and Wi-N for most cancer cells is 0.3–0.5 µM and >10–20 µM, respectively) [26,29,30]. The higher permeability coefficients of ARC and CAPE is due to the presence of hydrophobic diprenyl group and phenethyl group in their structures, respectively. The higher lipophilicity (XLogP3-AA in Table 2) of ARC and CAPE also implies that they undergo higher membrane retention compared to Wi-A and Wi-N. The relatively extreme negative PMF values of ARC within the membrane core surrounded by PMF peaks at lipid polar regions (Figure 6) implies that more ARC molecules will become trapped inside the membrane core. This might be an underlying cause of the low efficacy of ARC (IC50 = 275 µM) [58].

Wi-N, Wi-A and CAPE had notable differences in their permeability coefficients for the cancer and the normal cell membrane models, whereas ARC had closer values (Table 2). This implied that the differential distribution of PS in the membrane models does not have a notable influence on the permeation rate of ARC compared to that of Wi-A, Wi-N and CAPE. The permeability coefficients of Wi-N, Wi-A and CAPE were higher in cancer membranes compared to normal membranes, implying higher permeations rates due to the presence of PS in both leaflets (Table 2). The structural analogs Wi-N and Wi-A had similar fold differences in permeation rates between cancer and normal membranes (Table 2). As inferred from the PMF curves, the presence of PS in the outer leaflet lowered the free energy in the cell–water interface of the cancer membrane, aiding more molecules to bind to it. Hence, PS exposure may render higher tumor selectivity for Wi-N compared to Wi-A.

### 3.5. Implications of the Study

With the use of atomistic models and umbrella sampling methods to perform molecular dynamics simulations, the possible role of PS exposure in cancer cells in modulating the permeation of selected anti-cancer compounds have been interpreted. Several carrier systems using nanoparticles, synthetic polymers, liposomes, peptides and antibodies have been reported in the literature for targeted delivery of drugs to cancer cells [59,60,61,62,63,64]. However, successful clinical translations of such systems are limited. The present study relied on the fact that studying the molecules, which are inherently selective to cancer cells in action, will aid in understanding the molecular mechanisms that confer them selectivity. Though there are numerous factors that govern selectivity of a drug towards cancer cells, we have focused only on the effect of PS exposure on modulating membrane permeation. The exposure of PS caused alterations in the free energy landscapes underlying the traversal of the drug molecules through the membrane, thereby influencing the permeation. The molecular models and methods employed in this study have explained the selectivity of Wi-A, Wi-N and CAPE by differences in PMF landscapes and permeability coefficients between cancer and normal membrane models. In the case of ARC, PS exposure did not seem to have notable effects on the permeation rates. The interesting case is the structural analogs Wi-A and Wi-N, whose differential selectivity was explained by differences in free energy landscapes underlying membrane permeation. The used models demonstrate the possible contribution of PS exposure to drug selectivity. Hence, studying the influence of PS exposure on drug permeation offers, though not complete, a reasonable understanding of tumor selectivity of the drugs.

This study used simplistic molecular models to evaluate the possible contribution of PS exposure to drug selectivity and, hence, the limitations associated with the used materials and methods must be understood. The effect of PS exposure has been explored in the absence of other diverse types of lipid molecules, which might have led to a reduction in accuracy. The combination effect of different types of lipids and their distribution on the permeability of small molecules will differ from the observed sole effect of PS distribution in our binary lipid mixture. The models are not completely reflective of the cellular membrane, however, they aided in exploring one specific aspect that contributes to the differences in permeability between cancer and normal cells. The effect of PS exposure using complex models containing other major lipids will be explored in the future in light of the findings from this study. The proportion of charged species of small molecules in the tumor microenvironment and their interaction with the cell membrane also influence the permeation, and thereby their activity. Here, only the neutral species of the small molecules were studied.

## 4. Conclusions

Permeability of drugs through the cell membrane is crucial for its bioactivity. In this study, we have built cancer and normal membrane models based on PS distribution between membrane leaflets and assessed the permeation of natural compounds (Wi-A, Wi-N, CAPE and ARC) that have been shown to possess anti-cancer potential. It has been shown that PS exposure may influence drug permeation and thereby the drug activity. The cancer cell selectivity of these compounds was clearly evident. This study highlighted the effect of PS exposure in the cancer cell membrane on selective action of the chosen molecules. Though the simplistic models based on PS exposure might not be sufficient to explain the tumor selectivity of all drugs, this study explored a potential niche area, which may aid in the development or optimization of drugs that are inherently selective to cancer cells in action. With a high number of drugs failing in clinical trial due to lack of selectivity, the molecular differences between cancer and normal cell membranes can be exploited to improve the selectivity of potent drugs. Similar specialized in silico models of cancer cell membranes can be easily developed in the future to assess the tumor selectivity of existing drugs and to screen compound libraries to find cancer selective drugs.

## Figures and Tables

**Table 1:** The distribution of POPC and POPS molecules in cancer and normal membrane models.

Membrane	No. of POPC Molecules		No. of POPS Molecules	
	Outer Leaflet	Inner Leaflet	Outer Leaflet	Inner Leaflet
**Normal**	36	12	0	24
**Cancer**	24	24	12	12

**Table 2:** Calculated permeability coefficients of the drug molecules. Note: XLOGP3-AA indicates the computed octanol–water partition coefficients retrieved from the Pubchem database.

	Cancer Cell Membrane		Normal Cell Membrane		XLOGP3-AA
	*P* (cm/s)	*log P*	*P* (cm/s)	*log P*	
Withanone (Wi-N)	7.64 × 10^−6^	−5.12	1.33 × 10^−6^	−5.88	3.1
Withaferin A (Wi-A)	1.16 × 10^−3^	−2.94	1.06 × 10^−4^	−3.98	3.8
Caffeic Acid Phenethyl Ester (CAPE)	8.37 × 10^−1^	−0.08	2.31 × 10^−1^	−0.64	4.2
Artepillin C (ARC)	4.67	0.67	4.14	0.62	5.4

## Data Availability

The data generated during this study are included in this article.

## Supplementary Materials

The following supporting information can be downloaded at: https://www.mdpi.com/article/10.3390/membranes12010064/s1, Figure S1: PMF curves of Withaferin A (Wi-A), Withanone (Wi-N), Caffeic Acid Phenethyl Ester (CAPE) and Artepillin C (ARC) calculated after 10, 20, 30 and 40 ns: (A) PMF values in outer (extracellular) leaflet of normal membrane, (B) PMF values in inner (cytoplasmic) leaflet of normal membrane and (C) PMF values in outer leaflet of cancer membrane. Figure S2: PMF values of Withaferin A (Wi-A), Withanone (Wi-N), Caffeic Acid Phenethyl Ester (CAPE) and Artepillin C (ARC) before moving average smoothing: Magenta markers indicates PMF values in the cancer cell membrane model and cyan markers indicate PMF values in the normal cell membrane model. ‘0’ in horizontal axis indicates the center hydrophobic core of the membrane, negative values indicate the outer (extracellular) leaflet, positive values indicate the inner (cytoplasmic) leaflet. Error bars show standard deviation. Figure S3: Snapshots of last frame from umbrella sampling windows showing the converged orientations of Withaferin A (Wi-A) associated with permeation through (A–F) normal and (G–I) cancer membranes: Outer (extracellular) leaflet is shown at the top and inner (cytoplasmic) leaflet is shown at the bottom. (B,E) Orientations corresponding to the lowest points in PMF in the outer and inner leaflets of the normal membrane model. (H) Orientation corresponding to the lowest points in PMF in the leaflets of the cancer membrane model. Orientations are shown for only one leaflet in cancer membrane, as the cancer membrane model is symmetric. Colors are as per CPK rules. Figure S4: Snapshots of last frame from umbrella sampling windows showing the converged orientations of Withanone (Wi-N) associated with permeation through (A–F) normal and (G–I) cancer membranes: Outer (extracellular) leaflet is shown at the top and inner (cytoplasmic) leaflet is shown at the bottom. (B,E) Orientations corresponding to the lowest points in PMF in the outer and inner leaflets of the normal membrane model. (H) Orientation corresponding to the lowest points in PMF in the leaflets of the cancer membrane model. Orientations are shown for only one leaflet in cancer membrane, as the cancer membrane model is symmetric. Colors are as per CPK rules. Figure S5: Snapshots of last frame from umbrella sampling windows showing the converged orientations of Caffeic Acid Phenethyl Ester (CAPE) associated with permeation through (A–F) normal and (G–I) cancer membranes: Outer (extracellular) leaflet is shown at the top and inner (cytoplasmic) leaflet is shown at the bottom. (B,E) Orientations corresponding to the lowest points in PMF in the outer and inner leaflets of the normal membrane model. (H) Orientation corresponding to the lowest points in PMF in the leaflets of the cancer membrane model. Orientations are shown for only one leaflet in cancer membrane, as the cancer membrane model is symmetric. Colors are as per CPK rules. Figure S6: Snapshots of last frame from umbrella sampling windows showing the converged orientations of Artepillin C (ARC) associated with permeation through (A–F) normal and (G–I) cancer membranes: Outer (extracellular) leaflet is shown at the top and inner (cytoplasmic) leaflet is shown at the bottom. (B,E) Orientations corresponding to the lowest points in PMF in the outer and inner leaflets of the normal membrane model. (H) Orientation corresponding to the lowest points in PMF in the leaflets of the cancer membrane model. Orientations are shown for only one leaflet in cancer membrane, as the cancer membrane model is symmetric. Colors are as per CPK rules. Figure S7: Density of polar groups of cancer (magenta) and normal (normal) membranes along the reaction coordinate ‘z’. ‘0’ in the horizontal axis indicates the center hydrophobic core of the membrane, negative values indicate the outer (extracellular) leaflet, positive values indicate the inner (cytoplasmic) leaflet. Figure S8: Diffusivities of Withaferin A (Wi-A), Withanone (Wi-N), Caffeic Acid Phenethyl Ester (CAPE) and Artepillin C (ARC): Magenta markers indicates diffusivity values in the cancer cell membrane model and cyan markers indicate diffusivity values in the normal cell membrane model. ‘0’ in horizontal axis indicates the center hydrophobic core of the membrane, negative values indicate the outer (extracellular) leaflet, positive values indicate the inner (cytoplasmic) leaflet. Error bars show standard deviation.
