# Supplementary material for: Phosphatidylserine Exposed Lipid Bilayer Models for Understanding Cancer Cell Selectivity of Natural Compounds: A Molecular Dynamics Simulation Study
Source: Membranes (Basel). 2022 Jan 1;12(1):64. doi: 10.3390/membranes12010064 (PMC8780679; doi:10.3390/membranes12010064)

Supplementary File S1

Phosphatidylserine Exposed Lipid Bilayer Models for Understanding Cancer Cell Selectivity of Natural Compounds: A Molecular Dynamics Simulation Study

Navaneethan Radhakrishnan [1], Sunil C. Kaul [2], Renu Wadhwa [2,*] and Durai Sundar [1,3,*]

[1]DAILAB, Department of Biochemical Engineering & Biotechnology, Indian Institute of Technology (IIT) Delhi, Hauz Khas, New Delhi 110 016, India.
[2]AIST-INDIA DAILAB, DBT-AIST International Center for Translational & Environmental Research (DAICENTER), National Institute of Advanced Industrial Science & Technology (AIST), Tsukuba 305 8565, Japan
[3]School of Artificial Intelligence, Indian Institute of Technology (IIT) Delhi, New Delhi 110016, India

*Correspondence: renu-wadhwa@aist.go.jp (R.W), sundar@dbeb.iitd.ac.in (D.S.)

Figure S1: PMF curves of Withaferin A (Wi-A), Withanone (Wi-N), Caffeic Acid Phenethyl Ester (CAPE) and Artepillin C (ARC) calculated after 10, 20, 30 and 40 ns: (A) PMF values in outer (extracellular) leaflet of normal membrane, **(B)** PMF values in inner (cytoplasmic) leaflet of normal membrane and **(C)** PMF values in outer leaflet of cancer membrane.

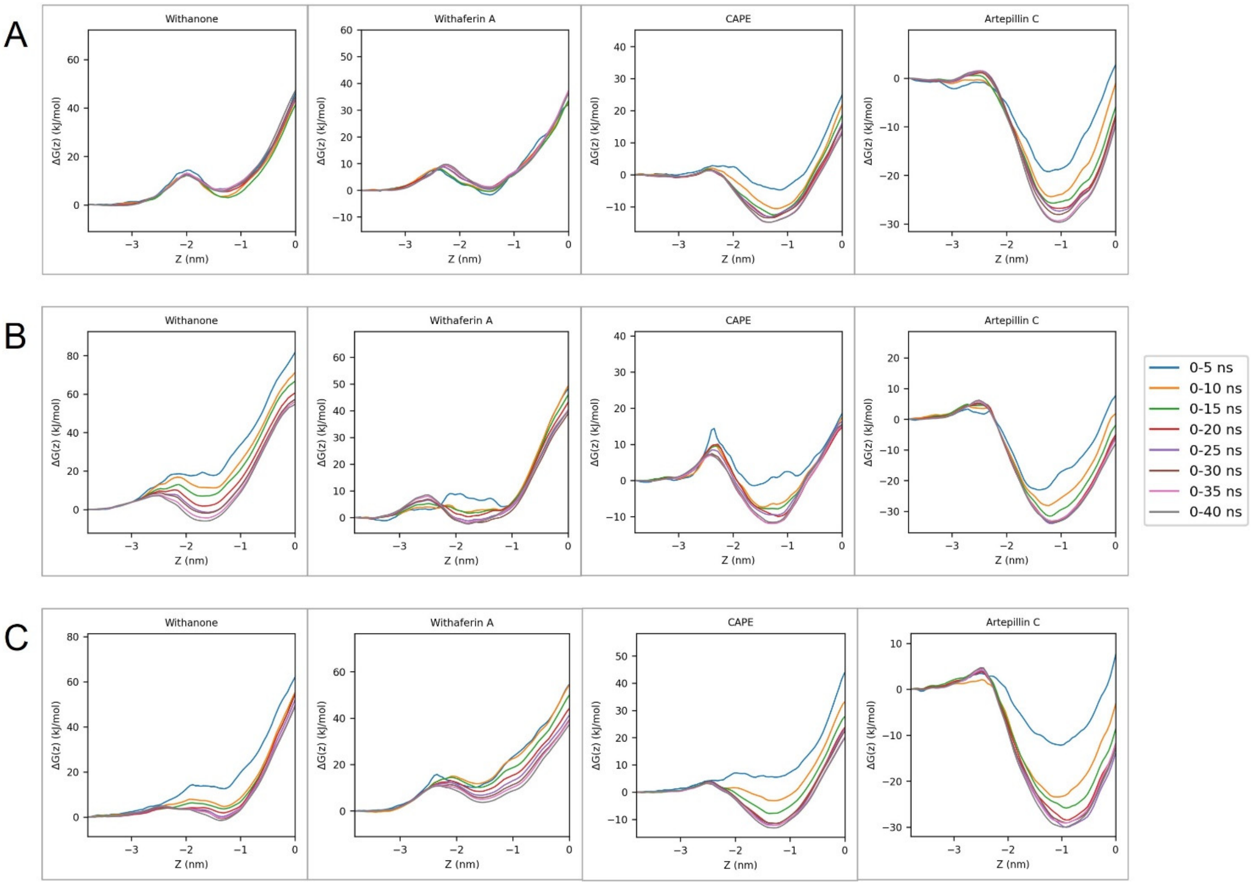

Figure S2: PMF values of Withaferin A (Wi-A), Withanone (Wi-N), Caffeic Acid Phenethyl Ester (CAPE) and Artepillin C (ARC) before moving average smoothing: Magenta markers indicates PMF values in the cancer cell membrane model and cyan markers indicate PMF values in the normal cell membrane model. '0' in horizontal axis indicates the center hydrophobic core of the membrane, negative values indicate the outer (extracellular) leaflet, positive values indicate the inner (cytoplasmic) leaflet. Error bars show standard deviation.

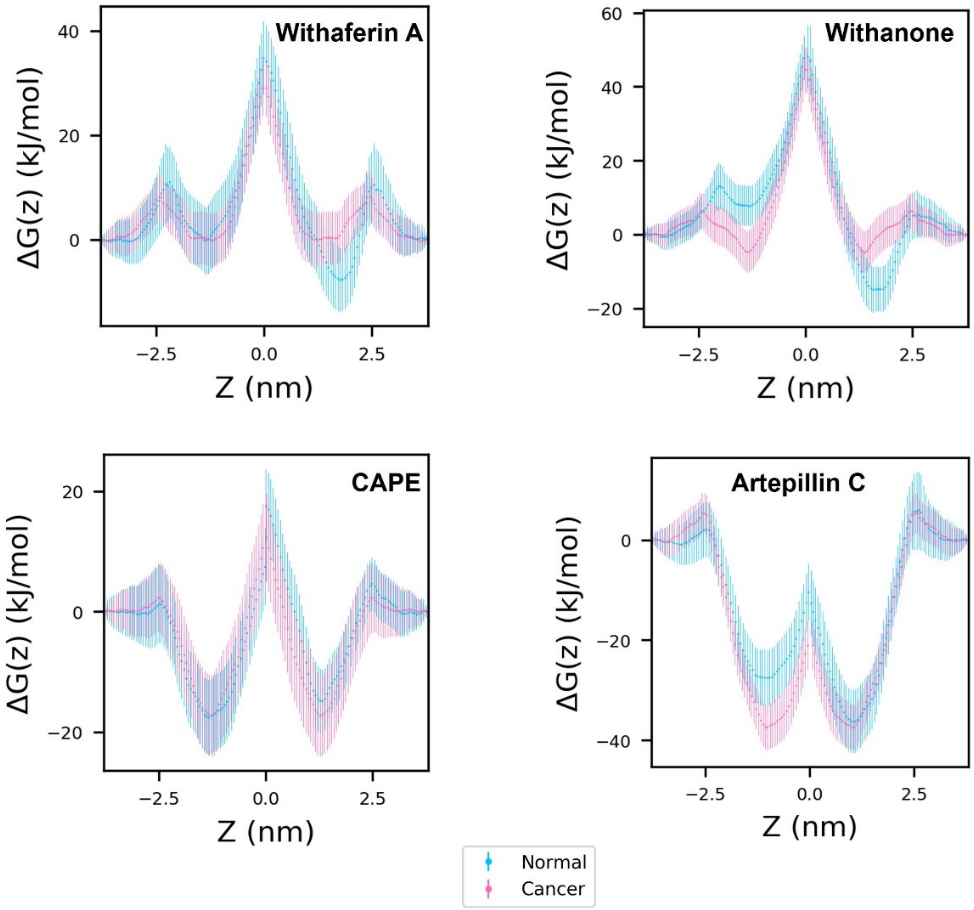

Figure S3: Snapshots of last frame from umbrella sampling windows showing the converged orientations of Withaferin A (Wi-A) associated with permeation through (A-F) normal and (G-I) cancer membranes: Outer (extracellular) leaflet is shown at the top and inner (cytoplasmic) leaflet is shown at the bottom. **(B,E)** Orientations corresponding to the lowest points in PMF in the outer and inner leaflets of the normal membrane model. **(H)** Orientation corresponding to the lowest points in PMF in the leaflets of the cancer membrane model. Orientations are shown for only one leaflet in cancer membrane, as the cancer membrane model is symmetric. Colors are as per CPK rules.

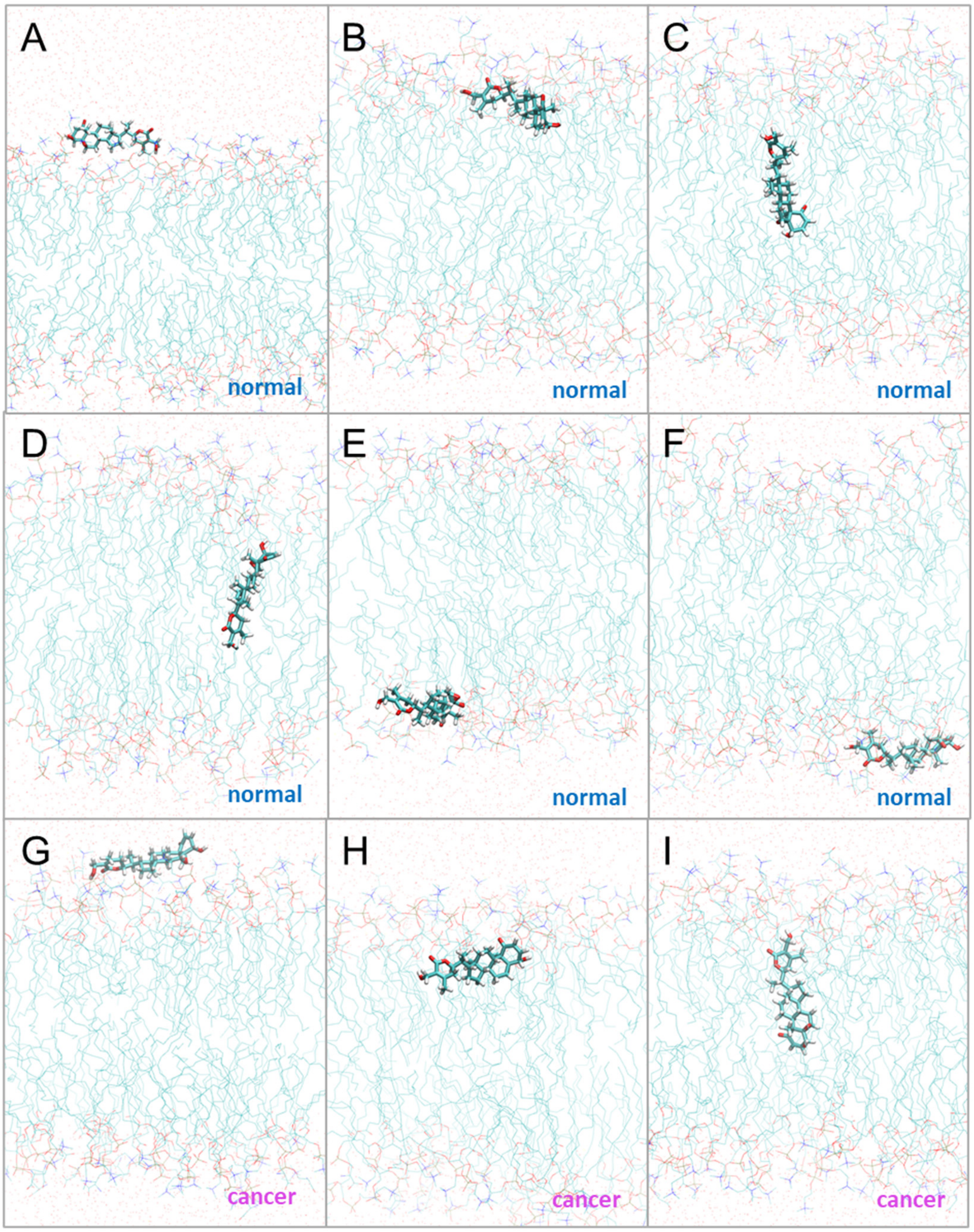

Figure S4: Snapshots of last frame from umbrella sampling windows showing the converged orientations of Withanone (Wi-N) associated with permeation through (A-F) normal and (G-I) cancer membranes: Outer (extracellular) leaflet is shown at the top and inner (cytoplasmic) leaflet is shown at the bottom. **(B,E)** Orientations corresponding to the lowest points in PMF in the outer and inner leaflets of the normal membrane model. **(H)** Orientation corresponding to the lowest points in PMF in the leaflets of the cancer membrane model. Orientations are shown for only one leaflet in cancer membrane, as the cancer membrane model is symmetric. Colors are as per CPK rules.

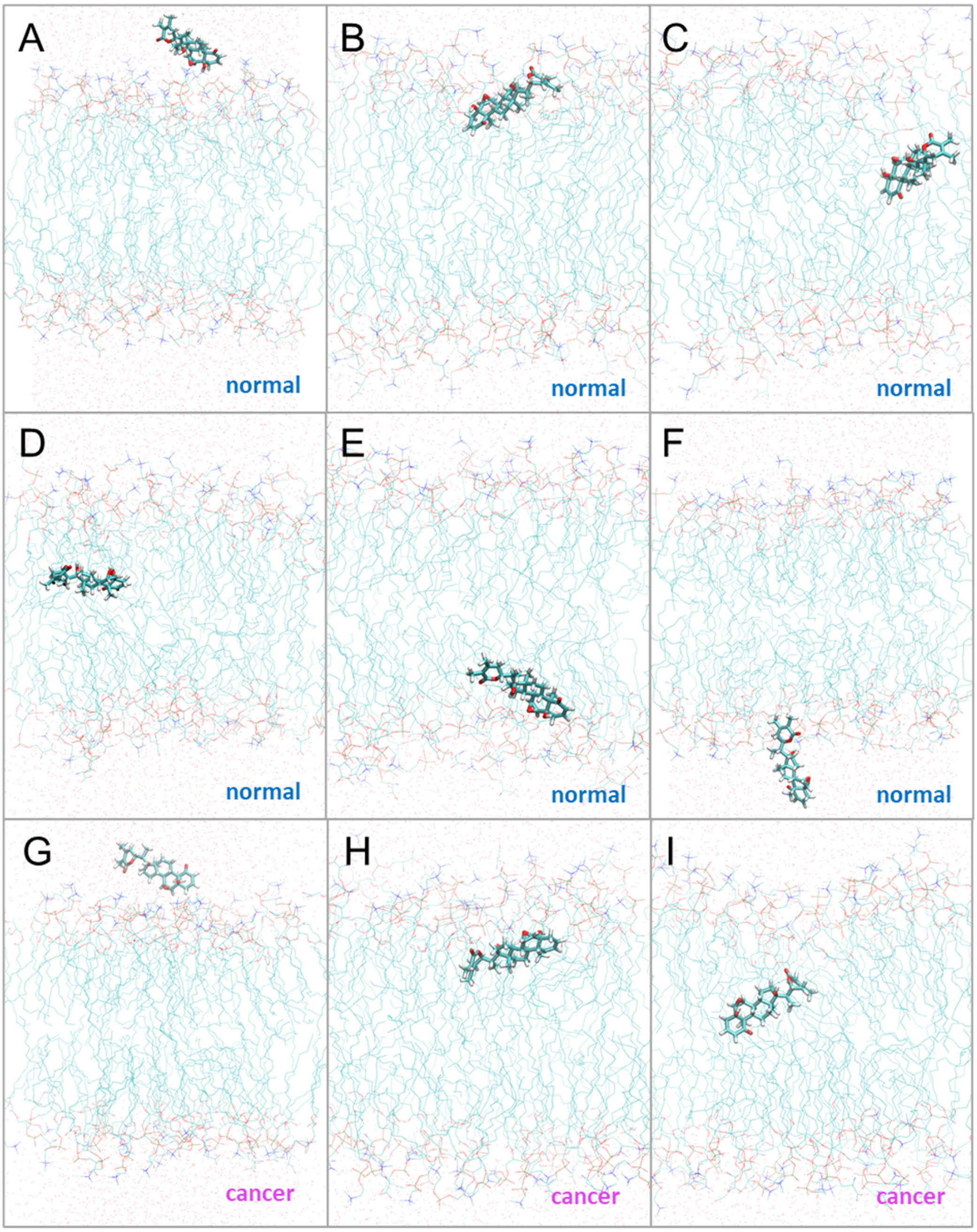

Figure S5: Snapshots of last frame from umbrella sampling windows showing the converged orientations of Caffeic Acid Phenethyl Ester (CAPE) associated with permeation through (A-F) normal and (G-I) cancer membranes: Outer (extracellular) leaflet is shown at the top and inner (cytoplasmic) leaflet is shown at the bottom. **(B,E)** Orientations corresponding to the lowest points in PMF in the outer and inner leaflets of the normal membrane model. **(H)** Orientation corresponding to the lowest points in PMF in the leaflets of the cancer membrane model. Orientations are shown for only one leaflet in cancer membrane, as the cancer membrane model is symmetric. Colors are as per CPK rules.

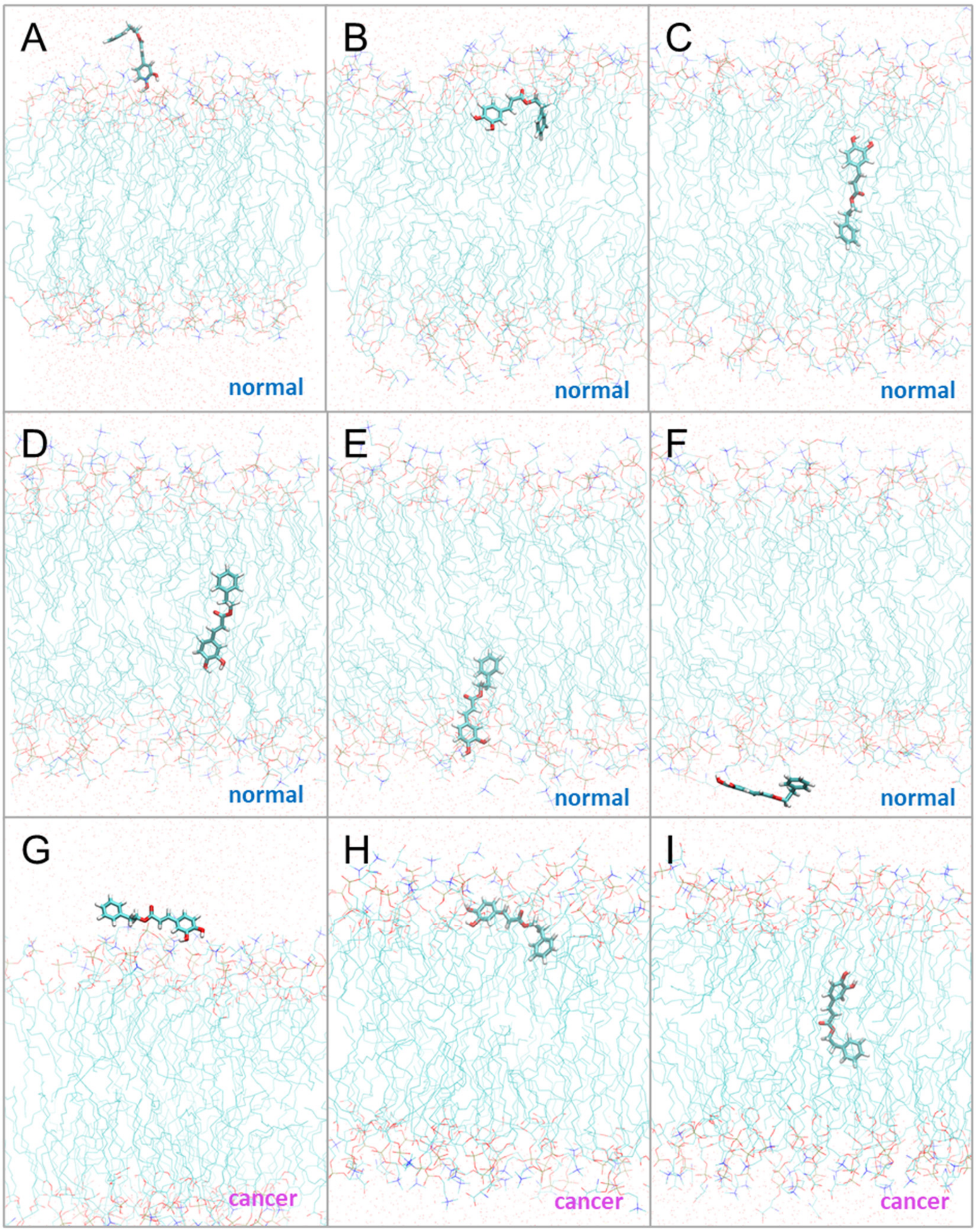

Figure S6: Snapshots of last frame from umbrella sampling windows showing the converged orientations of Artepillin C (ARC) associated with permeation through (A-F) normal and (G-I) cancer membranes: Outer (extracellular) leaflet is shown at the top and inner (cytoplasmic) leaflet is shown at the bottom. **(B,E)** Orientations corresponding to the lowest points in PMF in the outer and inner leaflets of the normal membrane model. **(H)** Orientation corresponding to the lowest points in PMF in the leaflets of the cancer membrane model. Orientations are shown for only one leaflet in cancer membrane, as the cancer membrane model is symmetric. Colors are as per CPK rules.

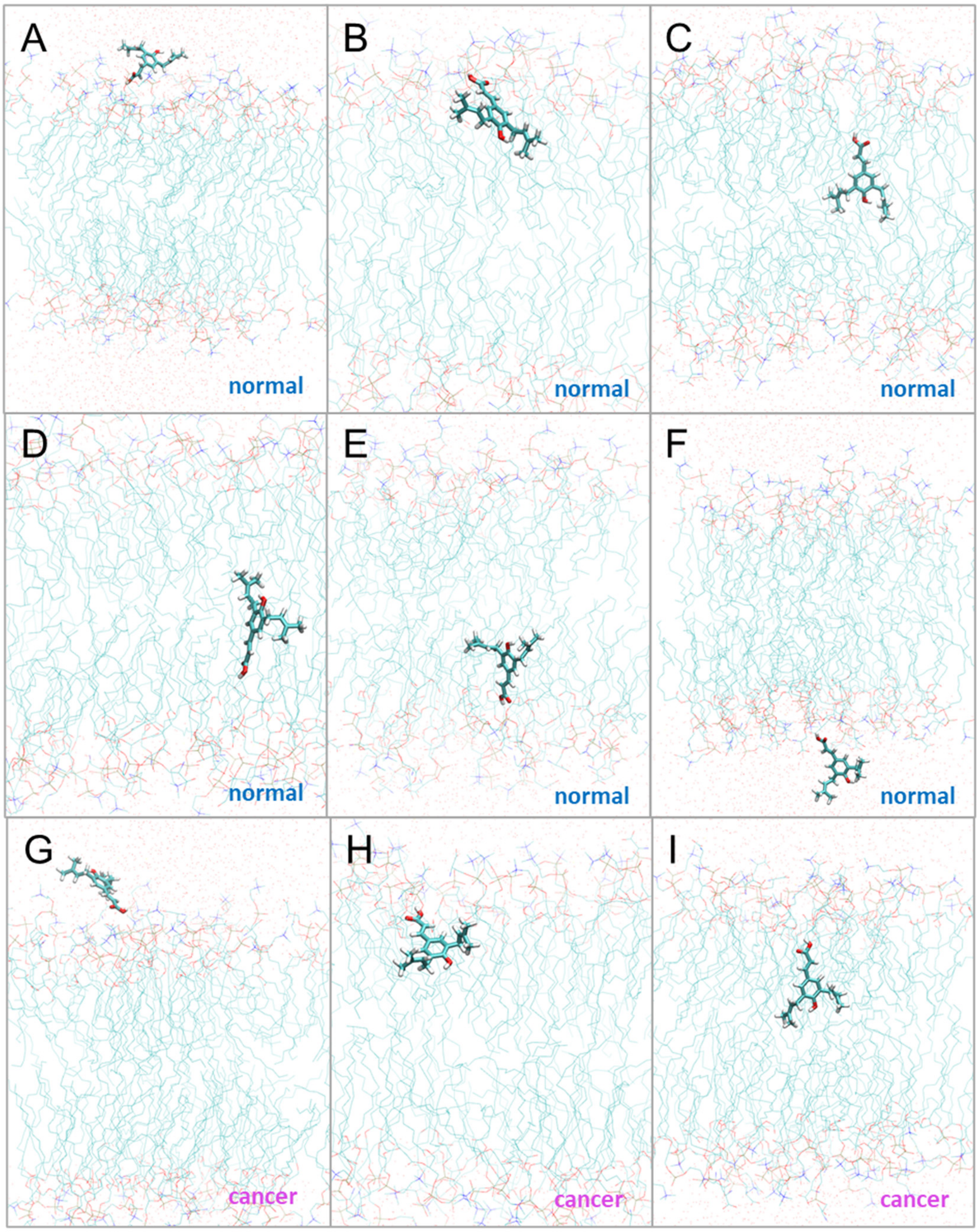

Figure S7: Density of polar groups of cancer (magenta) **and normal** (normal) **membranes along the reaction coordinate 'z'.** '0' in the horizontal axis indicates the center hydrophobic core of the membrane, negative values indicate the outer (extracellular) leaflet, positive values indicate the inner (cytoplasmic) leaflet.

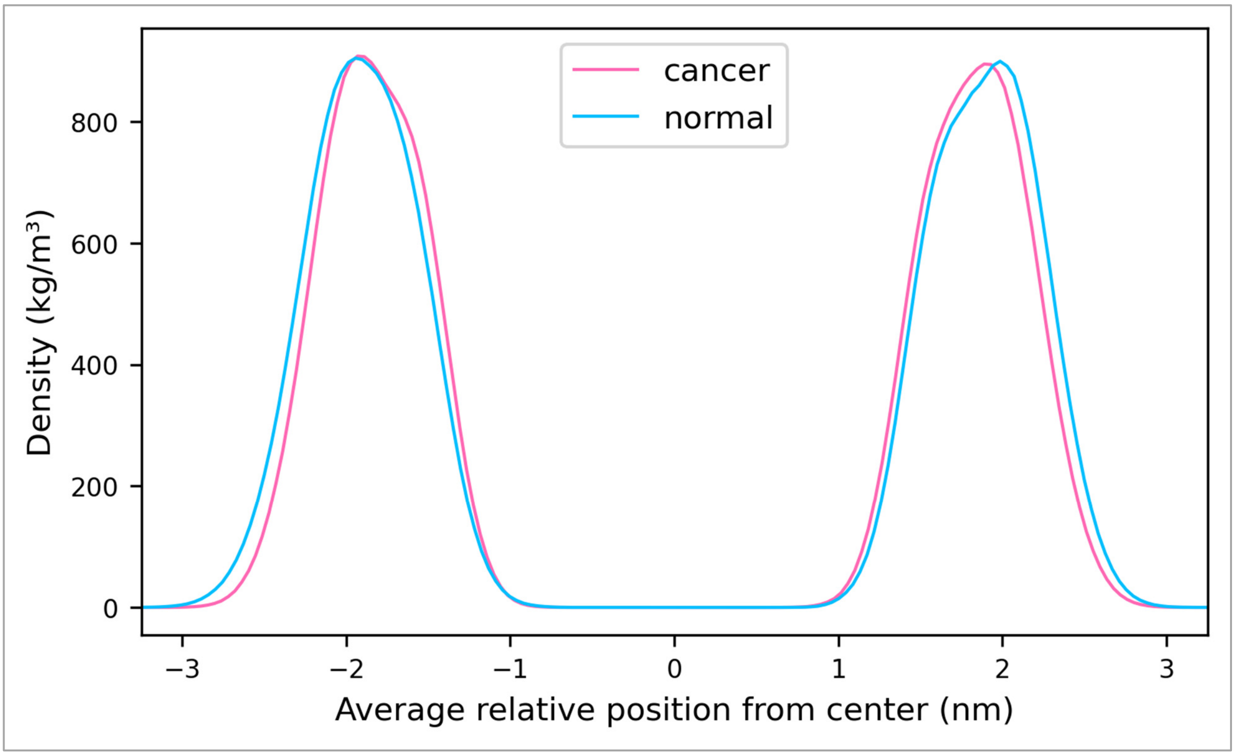

Figure S8: Diffusivities of Withaferin A (Wi-A), Withanone (Wi-N), Caffeic Acid Phenethyl Ester (CAPE) and Artepillin C (ARC): Magenta markers indicates diffusivity values in the cancer cell membrane model and cyan markers indicate diffusivity values in the normal cell membrane model. '0' in horizontal axis indicates the center hydrophobic core of the membrane, negative values indicate the outer (extracellular) leaflet, positive values indicate the inner (cytoplasmic) leaflet. Error bars show standard deviation.

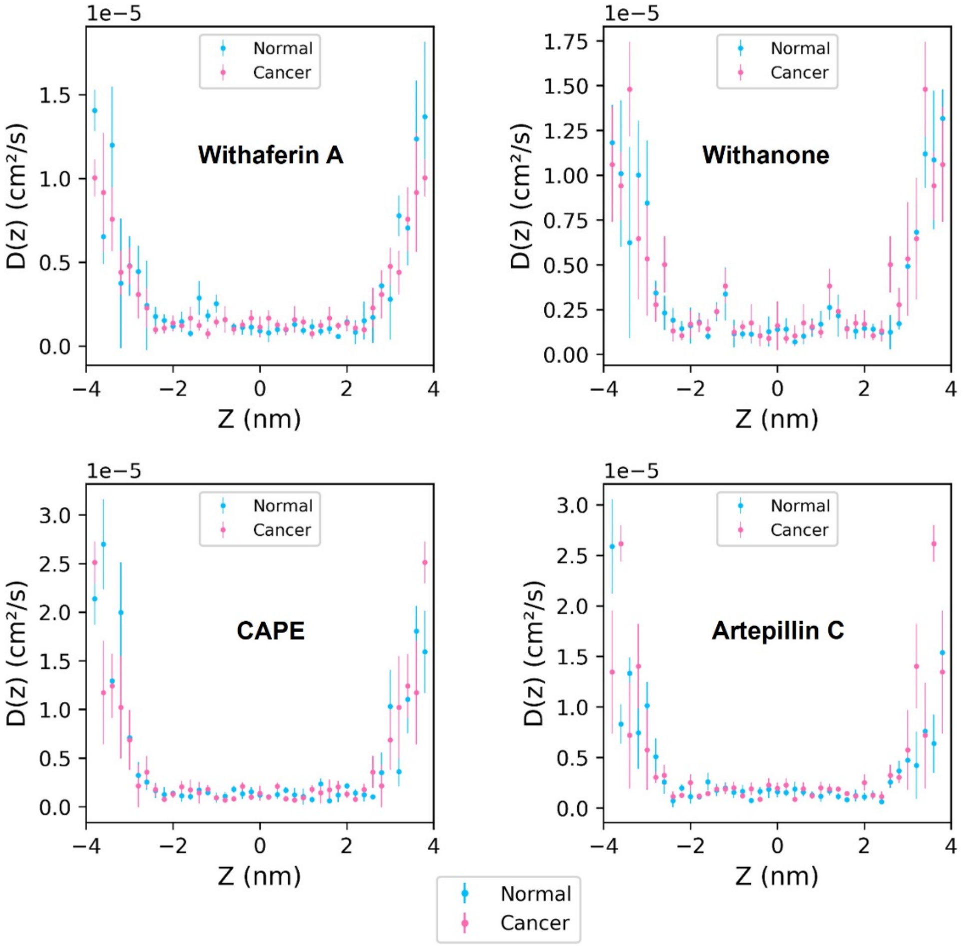

Supplement: Supplementary file 1 [file membranes-12-00064-s001.zip › membranes-1489670-supplementary.pdf]
